# Fluorescence *in situ* Hybridization method using Peptide Nucleic Acid probes for rapid detection of *Lactobacillus* and *Gardnerella* spp.

**DOI:** 10.1186/1471-2180-13-82

**Published:** 2013-04-12

**Authors:** António Machado, Carina Almeida, Débora Salgueiro, Ana Henriques, Mario Vaneechoutte, Freddy Haesebrouck, Maria João Vieira, Ligia Rodrigues, Nuno Filipe Azevedo, Nuno Cerca

**Affiliations:** 1 IBB - Institute for Biotechnology and Bioengineering, Centre of Biological Engineering, University of Minho, Campus de Gualtar, Braga, 4710-057, Portugal; 2 LEPAE, Department of Chemical Engineering, Faculty of Engineering, University of Porto, rua Dr. Roberto Frias, Porto, 4200-465, Portugal; 3 Laboratory of Bacteriology Research, Faculty Medicine & Health Sciences, University of Ghent, Ghent, B-9000, Belgium; 4 Laboratory of Veterinary Bacteriology and Mycology, Faculty of Veterinary Medicine, Ghent University, Merelbeke, B9820, Belgium

**Keywords:** Fluorescence *in situ* Hybridization (FISH), Peptide Nucleic Acid Probe (PNA probe), *Lactobacillus* spp., *Gardnerella vaginalis*, Bacterial vaginosis

## Abstract

**Background:**

Bacterial vaginosis (BV) is a common vaginal infection occurring in women of reproductive age. It is widely accepted that the microbial switch from normal microflora to BV is characterized by a decrease in vaginal colonization by *Lactobacillus* species together with an increase of *Gardnerella vaginalis* and other anaerobes. Our goal was to develop and optimize a novel Peptide Nucleic Acid (PNA) Fluorescence *in situ* Hybridization assay (PNA FISH) for the detection of *Lactobacillus* spp. and *G. vaginalis* in mixed samples.

**Results:**

Therefore, we evaluated and validated two specific PNA probes by using 36 representative *Lactobacillus* strains, 22 representative *G. vaginalis* strains and 27 other taxonomically related or pathogenic bacterial strains commonly found in vaginal samples. The probes were also tested at different concentrations of *G. vaginalis* and *Lactobacillus* species *in vitro,* in the presence of a HeLa cell line. Specificity and sensitivity of the PNA probes were found to be 98.0% (95% confidence interval (CI), from 87.8 to 99.9%) and 100% (95% CI, from 88.0 to 100.0%), for *Lactobacillus* spp.; and 100% (95% CI, from 92.8 to 100%) and 100% (95% CI, from 81.5 to 100.0%) for *G. vaginalis*. Moreover, the probes were evaluated in mixed samples mimicking women with BV or normal vaginal microflora, demonstrating efficiency and applicability of our PNA FISH.

**Conclusions:**

This quick method accurately detects *Lactobacillus* spp. and *G. vaginalis* species in mixed samples, thus enabling efficient evaluation of the two bacterial groups, most frequently encountered in the vagina.

## Background

Bacterial vaginosis (BV) is worldwide the most important perturbation of the normal vaginal condition in women [1]. BV affects pregnant women or women in reproductive age leading to high public health costs and associated complications, such as pelvic inflammatory disease, preterm birth, postoperative infections and an increased risk of acquisition and transmission of sexually transmitted diseases, such as human immunodeficiency virus (HIV) and human papillomavirus (HPV) [1,2]. Several studies have associated this condition with an imbalance in the vaginal microflora although BV etiology is still unclear [3-5]. BV has been described as a complex interaction of multiple factors related with several components of the vaginal microbial ecosystem and their human host, although many of these factors remain uncharacterized [2,6]. When BV is established, a decrease in the beneficial bacteria number, specifically *Lactobacillus* spp., and an increase in the numbers of anaerobic bacteria, such as *Gardnerella vaginalis*, *Atopobium vaginae* and *Mobiluncus* spp., are observed in the vaginal epithelium [7,8]. The disruption of the normal microflora and overgrowth by anaerobes are responsible for the BV signs and symptoms, namely the increase in vaginal pH (pH ≥ 4.5), the formation of vaginal biofilms on vaginal epithelia, observable as clue cells [9], fishy odor and milky vaginal discharge in the absence of an inflammatory response [9,10]. Although BV is often considered a polymicrobial condition, the predominant bacterial species identified is *G. vaginalis*[2]. In 2005, Swidsinski and colleagues characterized clinical vaginal swabs and found that a multispecies biofilm was formed, which was mainly composed of *G. vaginalis* and *Atopobium vaginae*. They hypothesized that *G. vaginalis* is the initial colonizing species and that its adherence is required before other BV-associated anaerobes are able to interact with the vaginal epithelium [10]. Due to *G. vaginalis* resistance against *Lactobacillus spp.* antimicrobial products, such as hydrogen peroxide and lactic acid [11,12], biofilm forming *G. vaginalis* might compete in initial adhesion against *Lactobacillus spp.* and may enable other anaerobes to incorporate and grow inside the biofilm [13]. Therefore, the development of an optimized and rapid diagnostic tool that allows the simultaneous visualization of *G. vaginalis* increase and *Lactobacillus* species reduction in vaginal samples could be of great use for further study of the previous hypothesis and as a diagnostic tool.

Fluorescence *in situ* Hybridization (FISH) is a molecular method used to identify and quantify microorganisms in a wide range of samples. This technique combines the simplicity of microscopic observation and the specificity of DNA/rRNA hybridization, allowing detection of selected bacterial species and morphologic visualization [14,15]. Nowadays, Peptide Nucleic Acid (PNA) probes are used instead of natural nucleic acids to improve FISH efficiency [16-19], because they enable more rapid and more specific hybridization [19-23]. The main goal of our work was to evaluate the PNA-FISH performance on mixed samples using a multiplex approach to detect *Lactobacillus* spp. and *G. vaginalis*. To validate the PNA probes, we determined, both *in silico* and *in vitro*, their specificity and sensitivity, using a broad diversity of representative *Lactobacillus* and *Gardnerella* strains, as well as other taxonomically related or pathogenic bacterial strains commonly found in vaginal samples. To confirm the usefulness of our methodology, the efficiency and specificity of the probes was also tested at different concentrations of *Lactobacillus* and *G. vaginalis* strains in the presence of a monolayer of HeLa cells.

## Methods

### Culture of bacterial strains

The bacterial strains used in this study are listed in Table 1. All strains from *Lactobacillus* spp. were grown in Man, Rogosa and Sharpe agar (MRS; Sigma, Portugal), excepting *L. iners* that was grown in Brucella Blood agar (BBA; Oxoid, United Kingdom) as well as *Atopobium vaginae* and *Gardnerella vaginalis*. The remaining bacterial species were cultured on Brain Heart Infusion agar (BHI; Oxoid, United Kingdom) or Trypticase Soy Agar (TSA; Oxoid, United Kingdom). Each bacterial culture was streaked onto fresh plates every 48–72 h. Plates were incubated at 37°C or 30°C (in the case of *L. pentosus* strains) under anaerobic conditions (AnaeroGen Atmosphere Generation system; Oxoid, United Kingdom) for 24–48 h prior to FISH experiments.

**Table 1:** Bacterial strains used in PNA-FISH assays and their specificity with Lac663 and Gard162 probes

**Bacterial species**	**Collection strain**	**Lac663 Probe efficiency**	**Gard162 Probe efficiency**
*Lactobacillus acidophilus*	ATCC 4356^T^	++++	-
*L. crispatus*	ATCC 33820^T^	++++	-
*L. gasseri*	ATCC 9857^T^	++++	-
*L. reuteri*	NCFB 2656^T^	+++	-
*L. rhamnosus*	ATCC 7469^T^	++++	-
*L. rhamnosus*	CECT 288^T^	++++	-
*L. johnsonii*	ATCC 11506^T^	++++	-
*L. hilgardii*	NCFB 962^T^	+++	-
*L. delbrueckii subsp. delbrueckii*	ATCC 9649^T^	+++	-
*L. delbrueckii subsp. lactis*	ATCC 12315^T^	+++	-
*L. pentosus*	CECT 4023^T^	++++	-
*L. casei*	CECT 5275^T^	++++	-
*L. coryniformis subsp. torquens*	CECT 4129^T^	++++	-
*L. paracasei*	CECT 227^T^	++++	-
*L. agilis*	CCUG 31450^T^	++++	-
*L. animalis*	ATCC 35046^T^	+++	-
*L. bifermentans*	ATCC 35409^T^	+++	-
*L. brevis*	ATCC 14869^T^	++++	-
*L. buchneri*	ATCC 4005^T^	+++	-
*L. fermentum*	ATCC 11739^T^	+++	-
*L. curvatus subsp. curvatus*	ATCC 25601^T^	++++	-
*L. farciminis*	DSM 20182T	++++	-
*L. fructivorans*	ATCC 8288^T^	+++	-
*L. gallinarum*	CCUG 31412^T^	++++	-
*L. graminis*	DSM 20719^T^	++	-
*L. hamsteri*	ATCC 43851^T^	+++	-
*L. helveticus*	ATCC 15009^T^	++++	-
*L. intestinalis*	ATCC 49335^T^	+++	-
*L. murinus*	ATCC 35020^T^	++++	-
*L. parabuchneri*	ATCC 12936^T^	++++	-
*L. paracasei subsp. paracasei*	CCUG 27320^T^	+++	-
*L. plantarum*	NCIMB 8827^T^	+++	-
*L. ruminis*	ATCC 27781^T^	++++	-
*L. sakei subsp. carnosus*	CCUG 8045^T^	++	-
*L. salivarius*	DEVRIESE 94/438^T^	+++	-
*L. plantarum*	NCCB 46043^T^	+++	-
*L. lactis* 53	-	-	-
*Streptococcus. thermophilus* A	-	-	-
*S. thermophilus* B	-	+++	-
*Leuconostoc mesenteroides*	-	-	-
*Bacillus subtilis*	DSM 7-10^T^	-	-
*Enterococcus faecium*	CECT 410^T^	-	-
*E. faecalis*	CECT 184^T^	-	-
*Gardnerella vaginalis 5-1*	-	-	++++
*G. vaginalis 101*	-	-	++++
*G. vaginalis AMD*	-	-	++++
*G. vaginalis*	ATCC	-	++++
*G. vaginalis*	Belgian isolate 1	-	+++
*G. vaginalis*	Belgian isolate 2	-	++++
*G. vaginalis*	Belgian isolate 3	-	++++
*G. vaginalis*	Belgian isolate 4	-	++++
*G. vaginalis*	Belgian isolate 5	-	++++
*G. vaginalis*	Belgian isolate 6	-	++++
*G. vaginalis*	Belgian isolate 7	-	+++
*G. vaginalis*	Belgian isolate 8	-	+++
*G. vaginalis*	Belgian isolate 9	-	++++
*G. vaginalis*	Belgian isolate 10	-	++
*G. vaginalis*	Belgian isolate 11	-	++++
*G. vaginalis*	Belgian isolate 12	-	+++
*G. vaginalis*	Belgian isolate 13	-	+++
*G. vaginalis*	Belgian isolate 14	-	++
*G. vaginalis*	Belgian isolate 15	-	+++
*G. vaginalis*	Belgian isolate 16	-	+++
*G. vaginalis*	Belgian isolate 17	-	++++
*G. vaginalis*	Belgian isolate 18	-	++++
*Atopobium vaginae*	CCUG 38953^T^	-	-
*A. vaginae*	CCUG 42099^T^	-	-
*A. vaginae*	CCUG 44116^T^	-	-
*A. vaginae*	Clinical isolate	-	-
*Bacillus cereus*	-	-	-
*Enterobacter aerogenes*	CECT 684^T^	-	-
*Escherichia coli O157:H7*	NCTC 12900^T^	-	-
*Staphylococcus aureus*	CECT 976^T^	-	-
*S. aureus*	CECT 86^T^	-	-
*Shigella flexneri*	ATCC 12022^T^	-	-
*Listeria monocytogenes*	-	-	-
*L. monocytogenes*	CECT 5873^T^	-	-
*L. seeligeri*	CECT 917^T^	-	-
*Klebsiella pneumoniae subsp. ozaenae*	ATCC 11296^T^	-	-
*Salmonella Typhi*	-	-	-
*S. enterica*	-	-	-
*Escherichia coli*	CECT 434^T^	-	-
*Prevotella bivia*	ATCC 29303^T^	-	-
*Mobiluncus mulieris*	ATCC 26-9^T^	-	-
*Fusobacteria nucleatum*	Clinical isolate	-	-

The PNA Probe (Lac663 and Gar162) efficiencies were tested in triplicate experiments for each strain, with the following hybridization PNA FISH qualitative evaluation: (−) Absence of hybridization; (++) Moderate hybridization; (+++) Good hybridization; (++++) Optimal hybridization. The table shows the median value from the three experiments for each strain.

### PNA probe design

To identify *Gardnerella* genus potential oligonucleotides-target for the probe design, we used the software Primrose [24], coupled with the 16S rRNA databases from the Ribosomal Database Project II (version 10.0; http://rdp.cme.msu.edu/) [25]. Complementarity with a low number of non-target and a high number of target sequences, as well as a higher predicted melting temperature and the absence of self-complementary sequences, were the main criteria for the PNA probe design. The selected sequences were synthesized (Panagene, Daejeon, South Korea) and the oligonucleotides N terminus was attached to an Alexa Fluor 594 molecule via a double 8-amino-3,6-dioxaoctanoic acid (AEEA) linker (PNA Probe: Gard162, Alexa Fluor 594-OO-CAGCATTACCACCCG; HPLC purified > 90%). The Gard162 probe hybridizes between positions 162 and 176 of the *G. vaginalis* strain 409–05 16S rRNA sequence (RDPII ID: S001872672) and was selected for probe design. For the detection of *Lactobacillus* spp. a previously developed probe [26], Lac663 was selected. This probe was attached to an Alexa Fluor 488 molecule, also via an AEEA linker (PNA Probe: Lac663, Alexa Fluor 488-OO-ACATGGAGTTCCACT; HPLC purified > 90%).

### *In silico* determination of sensitivity and specificity

Theoretical specificity and sensitivity were calculated according to Almeida *et al.*[27]. Briefly, the theoretical specificity and sensitivity of both probes were evaluated using updated databases available at the Ribosomal Database Project II (RDP II; http://rdp.cme.msu.edu/) through the Primrose software, and then were confirmed by a BLAST search at the National Centre for Biotechnology Information (http://www.ncbi.nlm.nih.gov/BLAST/; see Table 2). Only target sequences with at least 1200 base pairs and good quality were included. Briefly, theoretical sensitivity was calculated as *ts*/(*Tts*)×100, where *ts* stands for the number of target strains detected by the probe and *Tts* for the total number of target strains present in the RDP II database (http://rdp.cme.msu.edu/probematch/, last accession date, May 2012). Theoretical specificity was calculated as *nts*/(*Tnt*)×100, where *nts* stands for the number of non-target strains that did not react with the probe and *Tnt* for the total of non-target strains examined.

**Table 2:** **Theoretical specificity and sensitivity of several primers and probes for *Lactobacillus *and *Gardnerella *spp. detection**

**Probe**	**Type**	**Sequence (5´→3´)**	**No. of *Lactobacillus *strains detected **^**a**^	**No. of non- *Lactobacillus *strains detected **^**a**^	**Specificity (%)**^**a**^	**Sensibility (%)**^**a**^	**Reference or source**
Lab158^b^	DNA	GGTATTAGCA(C/T)CTGTTTCCA	11,991	7,165	99.30^g^	92.69 ^g^	[28]
LGC354A^c^	DNA	TGGAAGATTCCCTACTGC	12,701	12,329	98.79 ^g^	98.18 ^g^	[29]
LAB759^e^	DNA	CTACCCATRCTTTCGAGCC	10,371	2,823	99.72 ^g^	80.17 ^g^	[30]
Name not available	PNA	CCATTGTGGAAGATTC	12,930	14,880	98.54 ^g^	99.95 ^g^	[31]
Lac663	PNA	ACATGGAGTTCCACT	11,837	3,548	99.65 ^g^	91.50 ^g^	[26]
GardV	DNA	CCACCGTTACACCGAGAA	20	39	99.99	50.00	[10]
G.vag1008^f^	DNA	CTGCAGAGATGTGGTTTCCYTTCG	39	7	100.00	97.50	[32]
G.vag198	DNA	CCACTAAACACTTTCCCAACAAGA	34	0	100.00	85.00	[6]
GV003	DNA	AGACGGCTCCATCCCAAAAGGGTT	32	0	100.00	80.00	[33]
Gard162	PNA	CAGCATTACCACCCG	38	1	100.00	95.00	This work

^*a*^ Calculated through ProbeMatch/, last accession, May 2012) with the following data set options: Strain – Both; Source – Both; Size – > 1200 bp; Quality – Both.

^*b*^ DNA probe that also detects members of *Enterococcus*, *Pediococcus*, *Weissella*, *Vagococcus*, *Leuconostoc* and *Oenococcus spp.* used by Lebeer et al. [34].

^*c*^ DNA probe mainly detecting members of Lactobacillales and Bacillales, such as Lactobacillus spp., used in Olsen et al. [35].

^*e*^ DNA probe also detects members of *Ruminococcaceae sp. and Pediococcus sp.* used in Quevedo et al. [36]; The R symbol of the DNA probe sequence may be Adenosine or Guanosine, therefore Quevedo et al. [36] used a degenerate base in the sequence of the DNA probe to detect *Lactobacillus* spp.

^*f*^ The Y symbol of the DNA probe sequence may be Cytidine or Thymidine, therefore Fredricks et al. [6] used a degenerate base in the sequence of the DNA probe to detect *G. vaginalis*

^*g*^ Values determined in Machado et al.[26].

### FISH hybridization procedure

Biomass from a single colony of each strain was diluted and homogenised in sterile water, and then 20 μL were spread on epoxy coated microscope glass slides (Thermo Scientific, USA). For mixed samples (see Table 3), 10 μL of the final suspension from each strain suspension (prepared as previously referred) for the selected mixed sample were spread on glass slides. The slides were air-dried prior to fixation. Next, the smears were immersed in 4% (wt/vol) paraformaldehyde (Fisher Scientific, United Kingdom) followed by 50% (vol/vol) ethanol (Fisher Scientific, United Kingdom) for 10 min at room temperature on each solution. After the fixation step, the samples were covered with 20 μL of hybridization solution containing 10% (wt/vol) dextran sulphate (Fisher Scientific, United Kingdom), 10 mM NaCl (Sigma, Germany), 30% (vol/vol) formamide (Fisher Scientific, United Kingdom), 0.1% (wt/vol) sodium pyrophosphate (Fisher Scientific, United Kingdom), 0.2% (wt/vol) polyvinylpyrrolidone (Sigma, Germany), 0.2% (wt/vol) ficoll (Sigma, Germany), 5 mM disodium EDTA (Sigma, Germany), 0.1% (vol/vol) triton X-100 (Sigma), 50 mM Tris-HCl (at pH 7.5; Sigma, Germany) and 200 nM of the PNA probe. Subsequently, the samples on glass slides were covered with coverslips and incubated in moist chambers at the hybridization temperature under analysis (from 50°C to 72°C) during a range of hybridization times (from 230 to 180 min). Next, the coverslips were removed and a washing step was performed by immersing the slides in a pre-warmed washing solution for 30 min at the same temperature of the hybridization step. This solution consisted of 5 mM Tris-base (Fisher Scientific, United Kingdom), 15 mM NaCl (Sigma, Germany) and 0.1% (vol/vol) triton X-100 (at pH 10; Sigma, Germany). Finally, the glass slides were allowed to air dry.

**Table 3:** Results of the Lac663 and Gard162 probes specificity test in artificial mixed samples

**Species in the artificial mixed samples**	**Bacteria strain collection codes**	**Multiplex PNA-FISH assay**	
		**Lac663 Probe efficiency**	**Gard162 Probe efficiency**
*L. pentosus;*	CECT 4023^T^; -	++++	++++
*G. vaginalis 51*			
*L. casei;*	CECT 5275^T^; -	++++	++++
*G. vaginalis 101*			
*L. rhamnosus;*	CECT 288^T^; -	++++	++++
*G. vaginalis AMD*			
*L. crispatus;*	ATCC 33820^T^; -	++++	++++
*G. vaginalis ATCC*			
*L. delbrueckii sub. delbrueckii; Atopobium vaginae*	ATCC 9649^T^; CCUG 38953^T^	+++	-
*L. acidophilus;*	ATCC 4356^T^; CCUG 42099^T^	++++	-
*A. vaginae*			
*L. gasseri;*	ATCC 9857^T^; CCUG 44116^T^	++++	-
*A. vaginae*			
*L. paracasei sub. paracasei;*	CCUG 27320^T^; -	+++	−/+
*L. lactis* 53			
*L. rhamnosus;*	ATCC 7469^T^; CECT 410^T^	++++	-
*E. faecium*			
*L. reuteri;*	NCFB 2656^T^;	+++	-
*E. coli O157:H7*	NCTC 12900^T^		
*S. aureus;*	CECT 976^T^; -	-	++++
*G. vaginalis 51*			
*Shigella;*	ATCC 12022^T^; -	-	++++
*G. vaginalis 101*			
*L. seeligeri;*	CECT 917^T^; -	-	++++
*G. vaginalis AMD*			
*E. aerogenes;*	CECT 684^T^; -	-	++++
*G. vaginalis ATCC*			
*L. pentosus;*	CECT 4023^T^;	++++	++++
*G. vaginalis ATCC;*	-;		
*E. faecalis*	CECT 184^T^		
*L. casei;*	CECT 5275^T^;	++++	++++
*G. vaginalis AMD;*	-;		
*A. vaginae*	CCUG 38953^T^		
*L. rhamnosus;*	CECT 288^T^;	++++	++++
*G. vaginalis 101;*	-;		
*A. vaginae*	CCUG 42099^T^		
*L. crispatus;*	ATCC 33820^T^;	++++	++++
*G. vaginalis 51;*	-;		
*A. vaginae*	CCUG 44116^T^		
*L. casei;*	CECT 5275^T^;	++++	-
*L. mesenteroides;*	-;		
*A. vaginae*	CCUG 38953^T^		

The PNA probe (Lac663 and Gar162) efficiencies were tested in triplicate experiments for each strain, with the following hybridization PNA FISH qualitative evaluation: (−) Absence of hybridization; (+) Poor hybridization; (+++) Good hybridization; (++++) Optimal hybridization. Median values from the three experiments for each strain are shown in the table.

A FISH procedure in suspension was developed and optimized according to the previous work of Almeida and colleagues [27,37] and to the results obtained for the FISH procedure on glass slides described above. Hybridization was performed at 60°C for 90 min and for washing (60°C for 30 min) and a fresh solution was prepared less than 24 h before use. The suspension samples were stored at 4°C in the dark for a maximum of 24 h before microscopic observation/visualization. Both hybridization procedures (in glass slides and in suspension) are able to detect lactobacilli and *G. vaginalis* strains. While glass slide hybridization is the more commonly used technique in analytical laboratories [27], hybridization in suspension is frequently used to avoid autofluorescence background in complex matrix samples, besides being the hybridization technique used in flow cytometry [27,37].

### Microscopic visualization

Prior to microscopy, one drop of non-fluorescent immersion oil (Merck, Germany) was added to either slides or filters and covered with coverslips. Microscopic visualization was performed using an Olympus BX51 (Olympus Portugal SA, Porto, Portugal) epifluorescence microscope equipped with a CCD camera (DP72; Olympus) and filters capable of detecting the two PNA probes (BP 470–490, FT500, LP 516 sensitive to the Alexa Fluor 488 molecule attached to the Lac663 probe and BP 530–550, FT 570, LP 591 sensitive to the Alexa Fluor 594 molecule attached to the Gard162 probe).

Other filters (such as BP 365–370, FT 400, LP 421) present in the microscope, that are not capable of detecting the probe fluorescent signal were used to confirm the absence of autofluorescence. In each experimental assay, a negative control was performed simultaneously in which all the steps described above were carried out, but where no probe was added in the hybridization step. All images were acquired using Olympus CellB software using a total magnification of × 1000.

### Experimental assessment of probe specificity and sensitivity

After the hybridization optimization, the specificity and sensitivity of the PNA Lac663 and Gard162 probes were tested using 36 representative strains from the genus *Lactobacillus*, 22 representative strains from *Gardnerella vaginalis* (the only species of the genus *Gardnerella*[4]) and 27 representative strains from other related genera (see Table 1), of which 16 belonged to the order Lactobacillales and the other are common pathogens usually found in clinical samples, specifically strains from the following genera: *Atopobium, Bacillus*, *Lactococcus*, *Enterobacter*, *Enterococcus*, *Escherichia*, *Fusobacterium*, *Klebsiella*, *Leuconostoc*, *Listeria*, *Mobiluncus*, *Prevotella*, *Salmonella*, *Shigella*, *Staphylococcus* and *Streptococcus*[38-40]. All experiments were performed in triplicate at identical conditions and the experimental specificity and sensitivity were calculated.

### Detection of *Lactobacillus* spp. and *G. vaginalis* adhered to HeLa cell line

The application of cellular lines is a standard procedure that has already been used to mimic vaginal epithelium at several *in vitro* studies [41-43]. So, HeLa epithelial cells (from American Tissue Culture Collection, ATCC) were cultured at 37°C, in 5% CO_2_ (vol/vol), in Dulbecco’s modified Eagle’s medium (DMEM; Quality Biological, USA) supplemented with 10% FBS (vol/vol) and 1 IU penicillin/streptomycin ml^−1^ (MediaTech, Germany). Aliquots of 1ml from HeLa epithelial cells were seeded into 24-well tissue culture plates (Frilabo, Portugal) containing glass slides (12 mm) at a density of 2×10^5^cells per well, and incubated at 37°C and 5% CO_2_ (vol/vol) until the formation of a cell monolayer. The cultures were fed with fresh media every 48 hours. Simultaneously, several *Lactobacillus* (*L. crispatus* and *L. iners*) strains and *G. vaginalis* strain 5–1 were grown in MRS broth and BHI broth as described above. Prior to the adhesion assay, these broth cultures were harvested by centrifugation (4,000 *g*, 12 min, at room temperature) and washed twice with sterile phosphate buffer saline (PBS). Several standard concentrations of the bacteria were prepared in eukaryotic cell media (DMEM) and the optical density at 600 nm was adjusted using a microplate reader (Tecan, Portugal). When a HeLa cell monolayer was obtained, the cells were washed twice with 500 μl of sterile PBS to remove non adhered cells and culture media. Next, aliquots of 250 μl of cell culture media with a known concentration of a *Lactobacillus* strain and *G. vaginalis* 5–1 strain (1×10^3^ to 1×10^9^ CFU/ml; see Table 4) were added to each well with the washed cell monolayer from the 24-well tissue culture plate. Then the 24-well tissue culture plate was incubated for 30 min at 37°C in anaerobic conditions and 120 rpm. Finally, each well of the incubated plate was carefully washed twice with 500 μl of sterile PBS to remove non-adherent bacteria. The glass slides containing the adhered bacteria and eukaryotic cells were fixed and hybridized with both PNA probes and observed in fluorescence microscopy, as referred above. An additional 4',6-diamidino-2-phenylindole (DAPI; Sigma, Portugal) staining step was done at the end of the hybridization procedure, covering each of the glass slides with 10 μl of DAPI for 5 min at room temperature in the dark, followed by immediate observation in the fluorescence microscope. All these assays were repeated three times, on separate days, with three fields of view assessed each time.

**Table 4:** **Efficiency of the *Lactobacillus *spp. and *G. vaginalis *detection in adhesion assays with HeLa cell line**

**Concentration of cells (CFU/ml)**		**Multiplex PNA-FISH assay**	
***L. crispatus***	***G. vaginalis 5-1***	**Lac663 Probe efficiency**	**Gard162 Probe efficiency**
1×10^9^	1×10^9^	+++	+++
1×10^5^	1×10^5^	+++	+++
1×10^3^	1×10^3^	++++	+++
***L. iners***	***G. vaginalis 5-1***	**Lac663 Probe efficiency**	**Gard162 Probe efficiency**
1×10^9^	1×10^9^	+++	+++
1×10^5^	1×10^5^	+++	+++
1×10^3^	1×10^3^	++	+++

The PNA probe (Lac663 and Gar162) efficiencies were tested in each sample with the following hybridization PNA FISH qualitative evaluation: (++) Moderate hybridization; (+++) Good hybridization; (++++) Optimal hybridization. The table shows the median value from the three experiments for each sample.

## Results

### *In silico* analysis of PNA probes

The Lac663 probe showed a theoretical sensitivity and specificity of 91.5% and 99.7%, respectively, which corroborates the previously reported values [26]. Actually, this publication shows that these probes match the best values of the existing *Lactobacillus* probes. Gard162 probe presented a theoretical sensitivity of 95.0% and specificity of 100%. The theoretical specificity and sensitivity of these two probes and those developed in other studies were calculated as previously described by Almeida *et al.*[27] and are listed in Table 2. ProbeMatch tool, from RPDII (http://rdp.cme.msu.edu/probematch/; last accession, May 2012), was used with the following data set options: Strain – Both; Source – Both; Size – > 1200 bp; Quality – Both. For *Lactobacillus* probes, the specificity and sensitivity values previously determined [26], were considered.

### FISH Protocol optimization and autofluorescence-related factors

**Figure 1:**
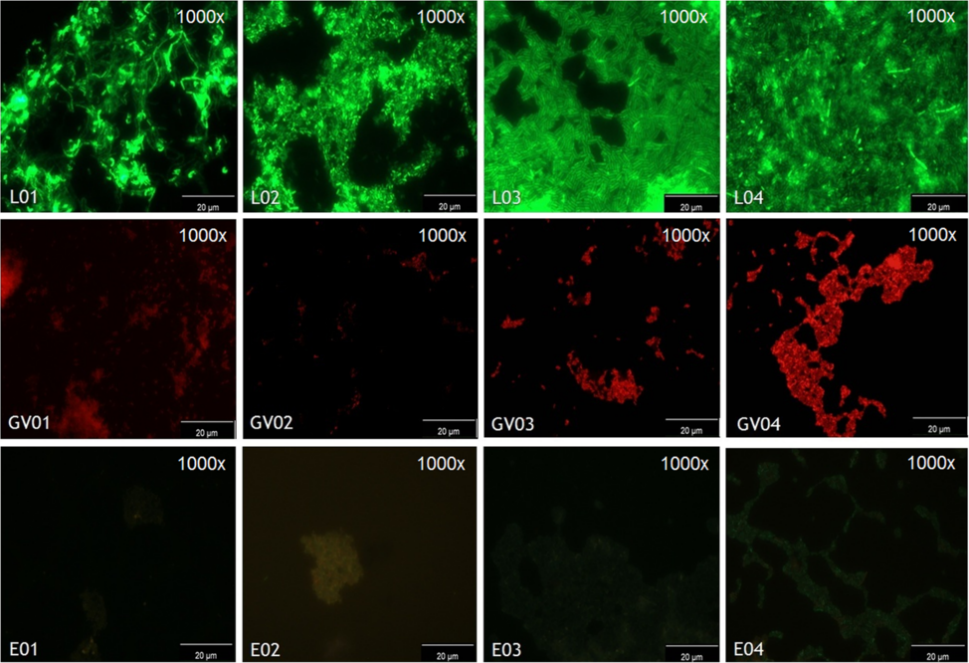
**Fluorescence microscopy pictures of *Lactobacillus *species, *G. vaginalis *and other related bacteria by PNA probes.** L01, *L. paracasei* CECT227; L02, *L. delbrueckii* ATCC9649; L03, *L. murinus* ATCC35020; L04, *L. salivarius* 438; GV01, *G. vaginalis* 5*–*1; GV02, *G. vaginalis* ATCC; GV03, Belgian *G. vaginalis isolate* 17; GV03, Belgian *G. vaginalis* isolate 18; E01, *Streptococcus thermophilus* A; E02, *Leuconostoc mesenteroides*; E03, *Enterococcus faecium*; E04, *Enterococcus faecalis*. The Lac663 and Gard162 PNA probes were associated with Alexa Fluor 488 and 594 fluorochromes, respectively.

FISH protocols on slides and in suspension were adapted from previous protocols developed by Almeida et al. [37], due to the crucial importance of fixation and hybridization conditions for an efficient multiplex FISH with different probes. From an initial temperature range of 50 to 72°C and an incubation time range between 30 and 180 min, the best hybridization conditions were set as a moist chamber temperature of 60°C during 90 min of incubation (*data not shown*). Hybridization conditions started to reveal strong signal-to-noise ratio at 59°C to 61°C from 30 min of incubation up to 120 min, reaching its peak at 60°C during 90 min of incubation. Hybridization conditions above 60°C and 90 min were also efficient, but the signal-to-noise ratio appeared to decrease beyond the selected values of time and temperature. Both hybridization protocols (on slides and in suspension) revealed the same results and pitfalls, as discussed below (some examples are shown in Figure 1).

### Experimental determination of probe specificity and sensitivity

As shown in Table 1, the Lac663 probe was able to detect all *Lactobacillus* strains and cross hybridization was found only for *Streptococcus thermophilus* B, as it was previously reported [26]. Based on these results, an experimental sensitivity of 100% (95% CI, 88.0 to 100.0%) and specificity of 98.0% (95% CI, 87.8 to 99.9%) were obtained for the Lac663 PNA probe. The Gard162 probe hybridized with all *G. vaginalis* strains, whereas no hybridization was observed for the other species tested. Therefore, this probe revealed a sensitivity of 100% (95% CI, 81.5 to 100.0%) and a specificity of 100% (95% CI, 92.8 to 100%).

### Detection of *Lactobacillus* spp. and *G. vaginalis* by Multiplex FISH

**Figure 2:**
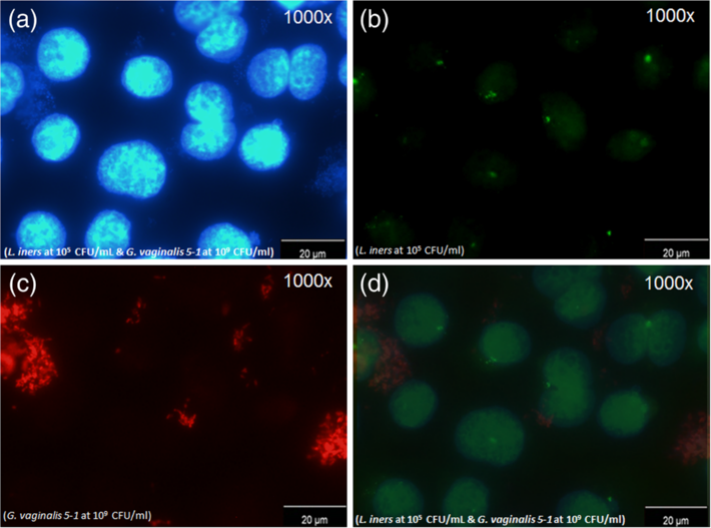
**Fluorescence microscopy pictures with *Lactobacillus *spp. and *G. vaginalis *at different concentrations against HeLa cell line. (a)**, blue filter; **(b)** green filter; **(c)** red filter; **(d)** overlay of the three previous filters. These fluorescence microscopy pictures were taken in the same microscopic field with *L*. *iners* and *G. vaginalis* 5–1 from culture strain collection at different concentrations against HeLa cell line by DAPI staining and specific PNA probes (Lac663 and Gard162), associated with Alexa Fluor 488 and 594 fluorochromes, respectively.

Once the hybridization procedure was fully optimized, the multiplex methodology was also tested against mixed bacterial cultures (containing *Lactobacillus* or/and *G. vaginalis* cells together with others species, see Table 3) and infected tissue cell line (Table 4). Lac663 and Gard162 probes selectively bound to *Lactobacillus* and *G. vaginalis* strains, respectively. The fluorescence signal was easily observable (Figure 2) and no cross hybridization with other species was detected (see Table 3). Additionally, the multiplex also performed well in the presence of HeLa cells (Table 4) for all the bacterial concentrations evaluated (1×10^3^ until 1×10^9^ CFU/ml), confirming the *in silico* analysis of the PNA probes previously elaborated.

## Discussion

### *In silico* and *in vitro* probe specificity and sensibility

Fluorescence microscopy has become a widely used technique for direct detection of bacteria in complex samples. In fact, many authors demonstrated the efficiency of FISH methodology for the analysis of lactobacilli and *G. vaginalis*[6,10,32,34,44-47]. However, the herein described multiplex approach may be the simpler to perform and still has high specificity for lactobacilli and *G. vaginalis* detection.

As shown in Table 1, the Lac663 and Gard162 probes bound highly specific to each target strain. Only Lac663 showed cross-hybridization with *S. thermophilus B*. However, *S. thermophilus* coccus morphology allows a clear differentiation from *Lactobacillus* spp., which has a rod-shaped morphology (with the exception of *L. iners*). Importantly, the Lac663 probe did not hybridize with several bacterial species from the *Bacilli* class and also with other common vaginal pathogenic bacteria, providing further evidence of its usefulness for *Lactobacillus* spp. detection in clinical samples.

Furthermore, the Gard162 probe showed hybridization with all *G. vaginalis* strains and no cross-hybridization was observed to other species, including other related pathogenic bacteria which may be present in the vaginal microflora, such as *A. vaginae*, *P. bivia*, *M. mulieris* and *F. nucleatum* (see Table 1). It is worth to mention that *in silico* analysis of the Gard162 probe only identified one non-target strain as match, more precisely *Bifidobacterium indicum* HM534842 (RDPII ID: S002908348). However, *B. indicum* is not a common bacterium from vaginal microflora, as it is usually present in the gut [48]. Recently a strong association between the bacterial loads in the vagina and rectum of pregnant women was described [49]. Although some gut bacteria such as *Escherichia coli*[48] have been associated with vaginal infections, *B. indicum* has not been described as a pathogenic bacterium [50]. The FISH efficiency and hybridization quality for the Gard162 probe, either alone or together with the Lac663 probe, confirmed the applicability of these two probes together in a multiplex PNA-FISH (see Figures 1 and 2).

As shown in Table 2, sensitivity and specificity equations allowed the comparison between our PNA probes and other published ones for *G. vaginalis* detection. For the *Lactobacillus* probe, this comparison had already been performed [26] and the Lac663 theoretical performance was found to be similar to other probes reported for *Lactobacillus* genus detection, but with a highest specificity. Also, Lab158, LGC354 and PNA Burton et al. [31] probes were found to cross-hybridize with one strain (RDPII ID: S000536416) from *G. vaginalis*, which might be incompatible with a multiplex approach to be used in vaginal samples. On the other hand, it is possible that this *G. vaginalis* strain was a misidentified *L. iners* strain, because confusion between both species has been reported [51].

Gard162 theoretical performance in specificity (100 %) was found to be similar to other probes for *G. vaginalis* detection that have been previously reported (Table 2). G.vag1008 is the only probe with higher sensitivity (97.5%) than our probe, being able to detect one more *G. vaginalis* strain. This higher sensitivity is due to the presence of a degenerate oligonucleotide in the sequence of the probe (see Table 2), allowing G.vag1008 to act as two different sequence probes. However, G.vag1008 has 24 oligonucleotides (i.e. 9 nucleotides more than our probe) and it is a DNA probe, which penetrates the cell wall less efficiently [52] and implies need for the use of long hybridization periods.

GardV probe detected species from several bacterial genera present in vaginal samples, such as *Alloscardovia*, *Parascardovia* and *Scardovia* spp. [53]. G.vag1008 probe hybridized with *Aeriscardovia* spp. that may also be found in vaginal samples [53] and therefore this represents an important pitfall for the *G. vaginalis* detection with such probes.

It is important to notice that our Gard162 probe is the first PNA probe specifically designed for *G. vaginalis* detection. Other PNA probes for the detection of lactobacilli [31,46] revealed several disadvantages when compared to Lac663 probe, as we shown before [26].

### Multiplex FISH detection

Although numerous authors attempted to correlate differences between healthy and BV vaginal samples [54-57], no consensus was achieved, except that biofilm formation of *G. vaginalis* and a decrease in lactobacilli number could be considered as the initial stages in the pathogenesis of BV [10,58]. Swidsinski and colleagues already conducted an international follow-up study in which vaginal samples from several BV patients were analyzed by DNA-based FISH and a dense as well as active bacterial biofilm on vaginal mucosa was detected, primarily consisting of *G. vaginalis*[47]. Therefore, multiplex FISH to analyze *G. vaginalis* biofilm establishment and subsequently lactobacilli replacement appeared to be a useful molecular methodology for BV diagnosis in vaginal samples. Although several authors already developed specific probes for *G. vaginalis* and *Lactobacillus* spp. detection for FISH, our multiplex method presented new improvements on the method (see Table 2).

Due to the difficulty to obtain fresh vaginal samples diagnosed with BV, we devised an *in vitro* experiment mimicking the shift from healthy vaginal flora to BV HeLa cells were incubated with different concentrations of *G. vaginalis* and *Lactobacillus* strains (*L. crispatus* and *L. iners*), ranging from normal to BV vaginal microflora contents (1×10^3^ to 1×10^9^ CFU/ml; see Table 4). The HeLa cell line is an established tool in experimental research with lactobacilli. It has not only been used to study attachment of several *Lactobacillus* species, but also of other pathogens [41-43]. The *Lactobacillus* strains used here were selected because high concentrations of *L. crispatus* (in conjugation with low loads or absence of *G. vaginalis*) are usually associated to the normal vaginal microflora while high concentrations of *L. iners* (in conjugation with high loads of *G. vaginalis*) are commonly associated to the microflora of BV diagnosed women [4,7,51]. The efficiency of our multiplex PNA-FISH methodology was demonstrated by ability of the PNA probes to hybridize in a large range of *Lactobacillus* spp. and *G. vaginalis* concentrations, even in the presence of epithelial cells (see Table 4). Swidsinski and colleagues [10,47] used a multiplex FISH methodology to study BV biofilms. A drawback of their approach is that it requires pre-treatment with lysozyme before fixation and the use of urine or paraffin-embedded samples, in opposition of our methodology that do not require a pre-treatment for FISH analysis. These experimental steps increase analysis time and decrease FISH efficiency for *Lactobacillus* spp. and *G. vaginalis* strains detection, due to the lower number of cells available for hybridization. Another DNA hybridization test for vaginal infection was studied by Witt and colleagues that evaluated the Affirm VPIII Kit [59], which detected *G. vaginalis*, *Candida* spp. and *Trichomonas vaginalis* in clinical samples, using two distinct single-stranded nucleic acid probes for each organism, which makes the analysis more complex and vulnerable to experimental pitfalls. This validated method showed sensitivity and specificity values for *G. vaginalis* of 89.5% and 97.1%, respectively, both lower than our Gard162 experimental values (95.0% and 100%, respectively). Furthermore, Fredricks and colleagues developed a FISH methodology for molecular identification of unknown bacteria associated with BV [6], using DNA probes Eub338-Cy5 and G.vag198-Cy3. However, the Eub338 is an unspecific probe used to detect *Lactobacillus* spp., detecting all species of the order Bacillales, and G.vag198 corresponds to a twenty five oligonucleotide probe with high specificity (100%) but with low sensitivity (85.0%) when compared to our probe (see Table 2). Both these probes worked together at a hybridization temperature of 45°C, which may easily lead to the occurrence of false positive results. Moreover, previous studies have shown that probes with Cy fluorochromes present a lower fluorescence signal than those with the corresponding Alexa Fluor [60].

To conclude, our main purpose was achieved by demonstrating the *in vitro* applicability of the PNA multiplex methodology for detection of *Lactobacillus* species and *G. vaginalis* in the presence of the HeLa epithelial cell line and other taxonomically related or pathogenic bacterial strains commonly found in vaginal samples. These *in vitro* results confirmed the previous *in silico* analysis from Lac663 and Gard162 probes.

## Conclusions

In summary, the use of the PNA multiplex FISH assay described here significantly increases the specificity and sensitivity of the detection of *Lactobacillus* spp. and *G. vaginalis* strains in mixed samples and no interference was observed in the presence of human epithelial cells. As previously referred, there are no consensual agreements regarding BV markers, except for lactobacilli number decrease and initial adherence, and consequent biofilm formation from *G. vaginalis*. Moreover, our approach allows a fast identification (approximately 3 hours) of the main bacteria involved in BV establishment. Further studies are necessary to detect BV biofilm formation in clinical samples and to characterize possible interactions with other unknown bacteria in the biofilm. The combination of our PNA-FISH methodology with EUB probe or other methodologies, such as electron microscopy, may help to better understand BV etiology.

## Competing interests

This work has been submitted as a patent.

## Authors’ contributions

AM, CA, DS and AH conceived of the study and participated in its design and drafted the manuscript. AM and CA carried out the PNA probes design and PNA-FISH assays. DS and AH worked in the PNA-FISH assays and HeLa cellular line culture, respectively. MV, FH, MJV and LR provided the bacteria culture collection for the study and helped to draft the manuscript. NFA and NC conceived of the study and participated in its design and coordination and helped to draft the manuscript. All authors read and approved the final manuscript.
